# Characteristics of Recent Generic Drug Approvals by the US Food and Drug Administration

**DOI:** 10.1001/jamanetworkopen.2019.13029

**Published:** 2019-10-11

**Authors:** Kuo Jiao, Ravi Gupta, Erin Fox, Aaron Kesselheim, Joseph S. Ross

**Affiliations:** 1 Yale University School of Public Health, New Haven, Connecticut; 2 Now with Fosun Pharma, Princeton, New Jersey; 3 Department of Medicine, Johns Hopkins Hospital, Johns Hopkins School of Medicine, Baltimore, Maryland; 4 University of Utah Health Care Drug Information Service, Department of Pharmacotherapy, University of Utah College of Pharmacy, Salt Lake City; 5 Program On Regulation, Therapeutics, And Law, Division of Pharmacoepidemiology and Pharmacoeconomics, Department of Medicine, Brigham and Women's Hospital, Harvard Medical School, Boston, Massachusetts; 6 Section of General Internal Medicine, Department of Medicine, Yale University School of Medicine, New Haven, Connecticut; 7 Department of Health Policy and Management, Yale University School of Public Health, New Haven, Connecticut; 8 Center for Outcomes Research and Evaluation, Yale New Haven Hospital, New Haven, Connecticut

## Abstract

This cross-sectional study describes the characteristics of abbreviated new drug applications approved by the US Food and Drug Administration, focusing on those with limited generic competition or with history of shortages.

## Introduction

Generic drugs have an essential role in the US health care system, providing less costly and equally safe and effective alternatives to brand-name drugs. Insufficient generic competition and shortages of older, off-patent drugs have disrupted care for patients and limited potential cost savings.^1,2^ In response, the US Food and Drug Administration (FDA) has taken a series of actions since 2017, including releasing the Drug Competition Action Plan^3^ and implementing certain aspects of the Generic Drug User Fee Amendments included in the FDA Reauthorization Act, to promote competition among drugs no longer protected by market exclusivity and expedite application review of generic drugs. The objective of this study was to characterize recent generic drug approvals, particularly for drugs that faced limited competition or had previously been in shortage.

## Methods

For this cross-sectional study, we used the Drugs@FDA database to identify all abbreviated new drug applications (ANDAs) approved by FDA from July 1, 2016, to December 31, 2018. We excluded tentative approvals, biologic treatments, over-the-counter products, and discontinued ANDAs. For each ANDA, we determined competition per FDA policy,^3^ categorized as the presence of 2 or fewer vs 3 or more existing manufacturers of the generic drug at the time of approval. Prior drug shortage was defined as any drug shortage lasting 1 month or longer for the same active ingredient and dosage from within 5 years prior to approval, determined using the University of Utah’s Drug Information Service drug shortage database.^1^ For each ANDA, we also characterized the corresponding brand-name drug’s initial approval year, priority review status, Orphan Drug Act designation, World Health Organization essential medicine status, therapeutic area based on World Health Organization Anatomical Therapeutic Chemical code classification, and drug complexity, the latter using previously described methods.^2^ We used descriptive statistics to characterize trends in recent generic drug approvals. We plotted quarterly trends from July 2016 to December 2018, including the proportion of ANDA approvals in each quarter (Q) that were drugs with limited competition and with prior drug shortage. We used SAS statistical software version 9.4 (SAS Institute) and Excel 2019 (Microsoft Corp) to conduct descriptive analyses without statistical testing. Because this study did not use patient data, it was exempt from review by the Yale Human Investiagtion Committee. Data analyses were conducted between March and August 2019. This study was reported using the Strengthening the Reporting of Observational Studies in Epidemiology (STROBE) reporting guideline.

## Results

There were 1832 ANDAs approved by the FDA from July 2016 to December 2018 (Figure), covering 533 different active ingredients. There were 133 ANDA approvals in Q3 2016, which initially increased over time before decreasing to 102 ANDA approvals in Q1 2018, but increased again to 262 ANDA approvals in Q4 2018. The corresponding brand-name drugs for these generic drugs were most commonly approved from 1995 to 2004 (761 drugs [41.5%]) and used to treat neurologic (426 drugs [23.3%]), cardiovascular (256 drugs [14.0%]), or infectious (229 drugs [12.5%]) diseases; 456 drugs (24.9%) received priority review, 244 drugs (13.3%) were designated as orphan drugs, 364 drugs (19.9%) were listed as World Health Organization essential medicines, and 351 drugs (19.2%) were determined to be complex drugs (Table).

**Figure.**
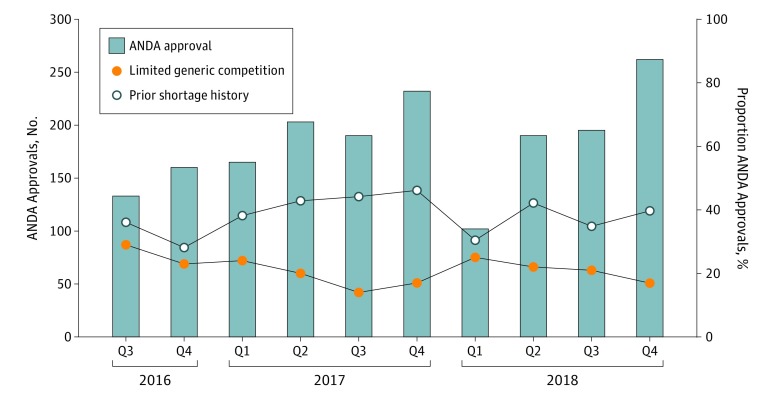
Generic Drug Approvals by the US Food and Drug Administration Overall and for Drugs With Limited Competition and Prior Shortages by Quarter (Q) From 2016 to 2018 Bars are graphed using the scale on the y-axis to the left; lines are graphed using the scale on the y-axis to the right. ANDA indicates abbreviated new drug applications.

**Table.** Characteristics of Generic Drugs Approved by the US Food and Drug Administration From July 2016 to December 2018

Characteristic	No. (%)
Generic competition at launch	
≤2 Approvals	374 (20.4)
≥3 Approvals	1458 (79.6)
Shortage within prior 5 y	
No	1115 (60.9)
Yes	717 (39.1)
Initial approval year	
Before 1984	414 (22.6)
1984-1994	283 (15.4)
1995-2004	761 (41.5)
2005-2015	374 (20.4)
Priority review	
Standard	1376 (75.1)
Priority	456 (24.9)
Orphan drug status^a^	
Standard	1588 (86.7)
Orphan	244 (13.3)
World Health Organization essential medicine^b^	
No	1468 (80.1)
Yes	364 (19.9)
Therapeutic area	
Alimentary tract and metabolism	150 (8.2)
Cardiovascular system	256 (14.0)
Dermatologic	157 (8.6)
Genitourinary system or sex hormones	151 (8.2)
Infectious disease	229 (12.5)
Blood disease or cancer	227 (12.4)
Nervous system or sensory organs	426 (23.3)
Other	236 (12.9)
Drug complexity^c^	
Noncomplex	1481 (80.8)
Complex	351 (19.2)

^a^ Orphan status is designated to drugs that treat rare diseases or conditions, defined as affecting fewer than 200 000 people in the United States.

^b^ Includes medications considered to be most effective and safe to meet the most important needs in a health system.

^c^ A drug product was considered complex if specific attributes make it difficult to manufacture the drug or establish equivalence, such as complex active ingredients (eg, peptides, complex mixtures, naturally sourced) or complex formulations (eg, colloids or liposomes).

Among 1832 ANDA approvals, 374 (20.4%) were in limited competition at the time of approval, ranging from 27 drugs (14.2%) in Q3 2017 to 39 drugs (29.3%) in Q3 2016. In addition, 717 drugs (39.1%) had experienced a shortage in the previous 5 years, ranging from 45 drugs (28.1%) in Q4 2016 to 107 drugs (46.1%) in Q4 2017.

## Discussion

From July 2016 to December 2018, the total number of generic drug applications approved by the FDA increased slightly, but the proportion of approvals for drugs that faced limited competition or had previously been in shortage remained steady. Our study has limitations, as we only characterized FDA approvals, not whether companies brought their approved drug to market after approval.^4^ Also, while we followed the FDA’s policy defining limited generic competition, prior studies have used a definition of 3 or fewer existing manufacturers.^5,6^ Although our results suggest that there have not yet been noticeable effects of the FDA’s initiatives to expand approvals for generic drugs at risk for price spikes and shortages, ANDAs take time for the manufacturer to prepare and then another 6 to 12 months for regulatory review. Nevertheless, continued attention is needed to foster approval of generic drugs with limited competition and prior shortage.

## References

[1] Chen SI, Fox ER, Hall MK, Despite federal legislation, shortages of drugs used in acute care settings remain persistent and prolonged. Health Aff (Millwood). 2016;35(5):-. doi:10.1377/hlthaff.2015.1157 27140985 PMC6712565

[2] Gupta R, Bollyky TJ, Cohen M, Ross JS, Kesselheim AS Affordability and availability of off-patent drugs in the United States: the case for importing from abroad: observational study. BMJ. 2018;360:k831. doi:10.1136/bmj.k831 29555641 PMC5858606

[3] US Food and Drug Administration FDA tackles drug competition to improve patient access. https://www.fda.gov/news-events/press-announcements/fda-tackles-drug-competition-improve-patient-access. Accessed April 10, 2019.

[4] Lupkin S, Hancock J Trump administration salutes parade of generic drug approvals, but hundreds aren’t for sale. https://khn.org/news/trump-administration-salutes-parade-of-generic-drug-approvals-but-hundreds-arent-for-sale/. Accessed April 10, 2019.

[5] Dave CV, Hartzema A, Kesselheim AS Prices of generic drugs associated with numbers of manufacturers. N Engl J Med. 2017;377(26):2597-2598. doi:10.1056/NEJMc1711899 29281576

[6] Gupta R, Kesselheim AS, Downing N, Greene J, Ross JS Generic drug approvals since the 1984 Hatch-Waxman Act. JAMA Intern Med. 2016;176(9):1391-1393. doi:10.1001/jamainternmed.2016.3411 27428055

